# Synthetic Methods of Phosphonopeptides

**DOI:** 10.3390/molecules25245894

**Published:** 2020-12-12

**Authors:** Jiaxi Xu

**Affiliations:** State Key Laboratory of Chemical Resource Engineering, Department of Organic Chemistry, College of Chemistry, Beijing University of Chemical Technology, Beijing 100029, China; jxxu@mail.buct.edu.cn; Tel./Fax: +86-10-6443-5565

**Keywords:** phosphonamidate, phosphonopeptide, β-phosphonopeptide, γ-phosphonopeptide, peptide

## Abstract

Phosphonopeptides are phosphorus analogues of peptides and have been widely applied as enzyme inhibitors and antigens to induce catalytic antibodies. Phosphonopeptides generally contain one aminoalkylphosphonic acid residue and include phosphonopeptides with C-terminal aminoalkylphosphonic acids and phosphonopeptides with a phosphonamidate bond. The phosphonamidate bond in the phosphonopeptides is generally formed via phosphonylation with phosphonochloridates, condensation with coupling reagents and enzymes, and phosphinylation followed by oxidation. Pseudo four-component condensation reaction of amides, aldehydes, alkyl dichlorophosphites, and amino/peptide esters is an alternative, convergent, and efficient strategy for synthesis of phosphonopeptides through simultaneous construction of aminoalkylphosphonic acids and formation of the phosphonamidate bond. This review focuses on the synthetic methods of phosphonopeptides containing a phosphonamidate bond.

## 1. Introduction

**Figure 1:**
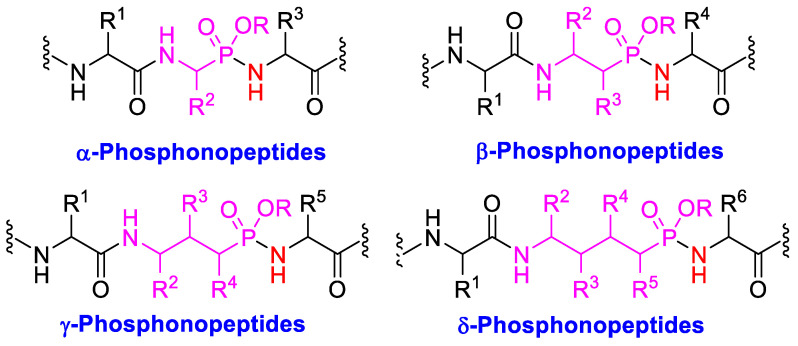
Different classes of phosphonopeptides.

Phosphonopeptides are phosphorus analogues of peptides. They generally contain one aminoalkylphosphonic acid residue and include phosphonopeptides with C-terminal aminoalkylphosphonic acids and phosphonopeptides containing a phosphonamidate bond [1,2]. Phosphonopeptides have been used as antibacterial agents [3]. They have been widely applied as enzyme inhibitors [4,5,6,7,8] and antigens for inducing catalytic antibodies [9,10,11,12] due to their tetrahedral structural feature. Phosphonopeptides containing C-terminal aminoalkylphosphonic acids have been prepared via coupling of *N*-protected amino acyl chlorides with aminoalkylphosphonic acids [13], condensation of *N*-protected amino acids or peptides and aminoalkylphosphonic acids with coupling reagents [14,15], aminolysis of *N*-chloroacetyl aminoalkylphosphonic acids [16], and the Mannich-type reactions of *N*-protected amino amides or aminoalkanesulfonamides, aldehydes, and phosphorus trichloride followed by hydrolysis [17,18]. Synthesis of phosphonopeptides with C-terminal aminoalkylphosphonic acids was reviewed recently [19]. This review focuses on the synthetic methods of phosphonopeptides, including α-, β-, γ-, and δ-phosphonopeptides (Figure 1), with a phosphonamidate bond, especially focuses on the synthetic strategies for the formation of the phosphonamidate bond, excluding the modification of phosphonopeptides.

## 2. Synthesis of Phosphonopeptides via Phosphonochloridates

Phosphonylation of amino/peptide esters with alkyl *N*-protected aminoalkylphosphonochloridates is a general and widely applied method for the synthesis of phosphonopeptides containing a phosphonamidate bond. The phosphonochloridates are usually prepared via chlorination of the corresponding dialkyl phosphonates with phosphorus pentachloride [13,20] or phosphorus oxychloride [21], chlorination of phosphonic monoesters with thionyl chloride [22,23] or oxalyl chloride [24,25], and chlorination of alkyl trimethylsilyl phosphonates [26] or alkyl phosphinates [27] with carbon tetrachloride. Phosphonobromidates are seldom applied as intermediates in the synthesis of phosphonopeptides and generated via bromination of alkyl phosphinates with bromine [28]. Each of the above-mentioned methods will be presented as following.

### 2.1. Chlorination of Dialkyl Phosphonates with Phosphorus Pentachloride

**Scheme 1:**
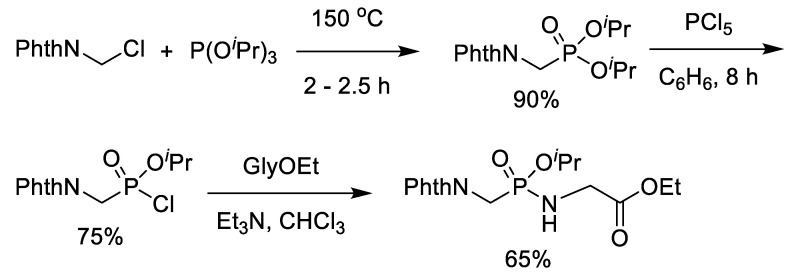
First synthesis of phosphonopeptide.

After aminomethylphosphonic acid was isolated from numerous organisms and animal and human organs [29,30], to understand the biological significance of this new class of compounds, phosphonodipeptide was synthesized in 1973. Diisopropyl *N*-phthalyl(Phth)aminomethylphosphonate was prepared from phthalylaminomethyl chloride and triisopropyl phosphite and further treated with phosphorus pentachloride to give the corresponding isopropyl *N*-phthalylaminomethylphosphonochloridate, which reacted with ethyl glycinate in the presence of triethylamine to give rise to protected phosphonopeptide (Scheme 1) [13]. This is the first chemical synthesis of α-phosphonopeptide with a phosphonamidate linkage. 

**Scheme 2:**
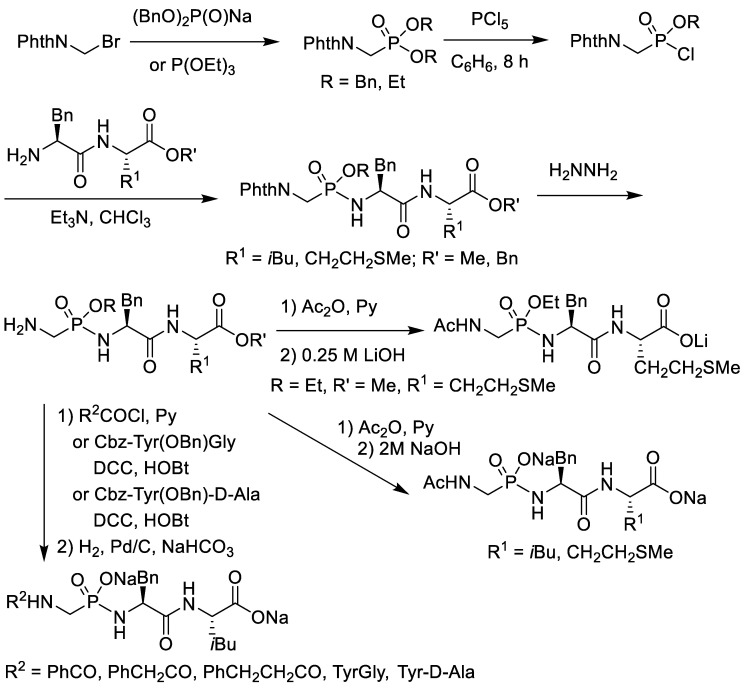
Synthesis of phosphonopeptides as inhibitors of enkephalinase and angiotensin-converting enzyme.

Similarly, *N*-phthalylaminomethylphosphonochloridate was prepared and further coupled with dipeptide esters to afford phosphonotripeptides. After hydrazinolysis and acetylation with acetic anhydride and acylation with acyl chlorides or *N*-benzyloxycarbonyl(Cbz)-protected dipeptides, the phosphonotripeptides were transformed into *N*-acetyl phosphonotripeptides, *N*-acyl phosphonotripeptides, and phosphonopentapeptides after hydrogenolysis, respectively (Scheme 2). As inhibitors of enkephalinase and angiotensin-converting enzyme (ACE), these phosphonopeptides exhibited good inhibitory potency against enkephalinase with several of the analogs having *K*_i_ values in the submicromolar range as contrasted to micromolar or higher toward ACE [20]. Another series of phosphonopeptides were synthesized as potential inhibitors of ACE [21]. The phosphonamide bond in the phosphonopeptides is generally stable under weak acidic and basic conditions.

### 2.2. Chlorination of Dialkyl Phosphonates with Phosphorus Oxychloride

**Scheme 3:**
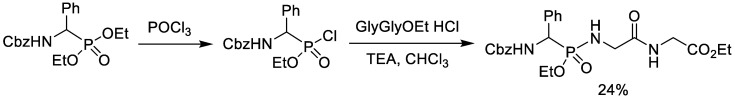
Synthesis of phosphonopeptide from phosphonochloridate generated by chlorination of diethyl phosphonate with phosphorus oxychloride.

Besides phosphorus pentachloride, phosphorus oxychloride was also applied in the conversion of dialkyl phosphonates into phosphonochloridates. Phosphonopeptide was synthesized from ethyl glycylglycinate hydroloride and ethyl phosphonochloridate derived from direct chlorination of diethyl phosphonate with phosphorus oxychloride (Scheme 3) [21].

### 2.3. Chlorination of Alkyl Phosphonic Acid Monoesters with Thionyl Chloride

**Scheme 4:**
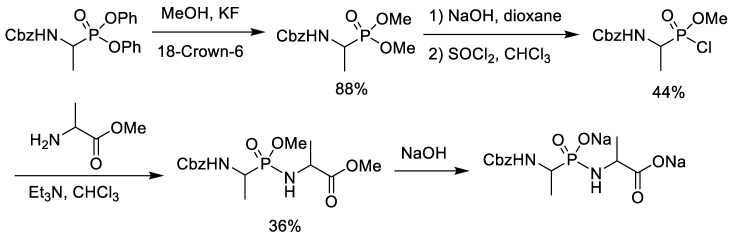
Synthesis of phosphonopeptide as the inhibitor of d-alanine:d-alanine ligase.

To search for competitive inhibitors of d-alanine:d-alanine ligase, phosphonodipeptide was prepared from methyl alaninate and *N*-Cbz-protected methyl 1-aminoethylphosphonochloridate, which was prepared from *N*-Cbz-protected diphenyl 1-aminoethylphosphonate via transesterification, selective hydrolysis, and chlorination with thionyl chloride (Scheme 4). The phosphonodipeptide was the competitive inhibitor of d-alanine:d-alanine ligase, with *K*_i_ close to 10^−6^ M [22]. 

**Scheme 5:**
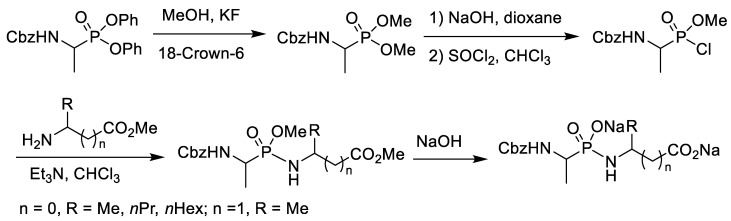
Synthesis of phosphonopeptides from aminoalkylphosphintes.

Some α-phosphonodipeptides which simulated the transition state of d-alanyl-d-alanine synthetase (EC 6.3.2.4) reaction were synthesized (Scheme 5). The inhibition of the synthetase by these phosphonopeptides was studied with the *S. faecalis* enzyme. The phosphonopeptides exhibited time-dependent inhibition in the presence of ATP, suggesting that they underwent phosphorylation prior to inactivating the enzyme [23].

**Scheme 6:**
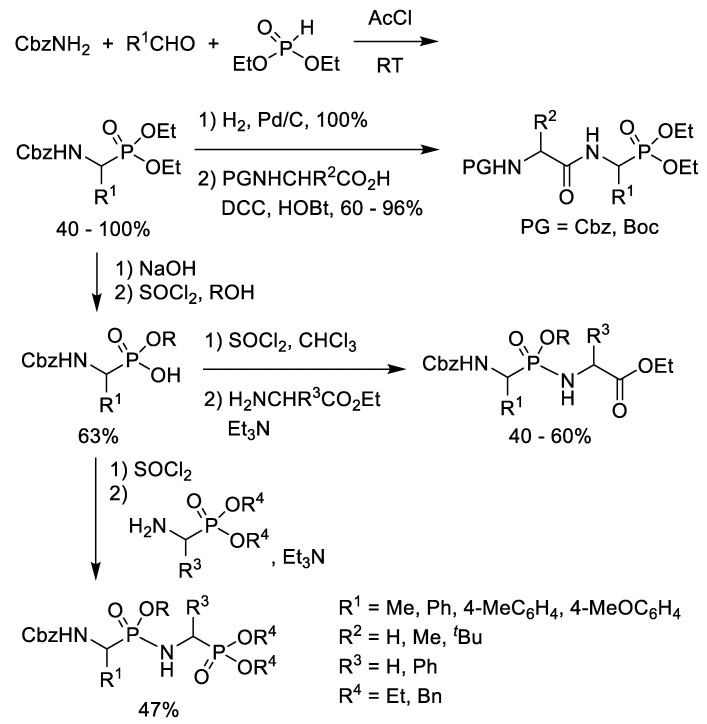
Synthesis of phosphonopeptides composing of one or two 1-aminoalkylphosphonic acid residue(s).

*N*-Cbz-protected diethyl 1-aminoalkylphosphonates were prepared via three component condensation reaction of benzyl carbamate, aldehydes, and diethyl phosphite in acetyl chloride. After hydrogenolysis and coupled with *N*-protected amino esters, they were converted into phosphonodipeptides with C-terminal 1-aminoalkylphosphonic acids. *N*-Cbz-protected diethyl 1-aminoalkylphosphonates were transformed to *N*-Cbz-protected 1-aminoalkylphosphonic monoesters via basic hydrolysis and treatment with thionyl chloride and alcohol. *N*-Cbz-protected 1-aminoalkylphosphonic monoesters were treated with thionyl chloride followed by reactions with amino esters or 1-aminoalkylphosphonates, affording phosphonopeptides composing of one or two 1-aminoalkylphosphonic acid residue(s) (Scheme 6) [31].

**Scheme 7:**
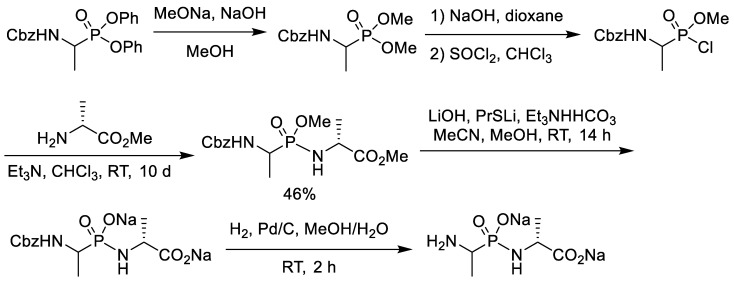
Synthesis of phosphonopeptide inhibitors of VanX.

To prepare novel inhibitors of VanX, a Zn(II) metalloenzyme that was required for high-level vancomycin resistance in bacteria, *N*-[(1-aminoethyl)hydroxyphosphinyl]-d-alanine was synthesized via coupling of *N*-Cbz protected methyl 1-aminoethylphosphonochloridate with methyl d-alaninate followed by basic hydrolysis and hydrogenolysis (Scheme 7). Bioassay results indicated that phosphonodipeptide was shown to be a partial competitive inhibitor of VanX with a *K*i of 36 μM [7,32].

Phosphonopeptides CbzNHCH_2_P(O)(OEt)-l-ProOBn, CbzNHCH_2_P(O)(OEt)-d-Pro, CbzNHCH_2_P(O)(OEt)-l-thioProOMe, and H_2_NCH_2_P(O)(OEt)-l-Pro were prepared following similar method for investigation on the relative catalytic efficiency of β-lactamase catalyzed acyl and phosphyl transfer [33].

**Scheme 8:**
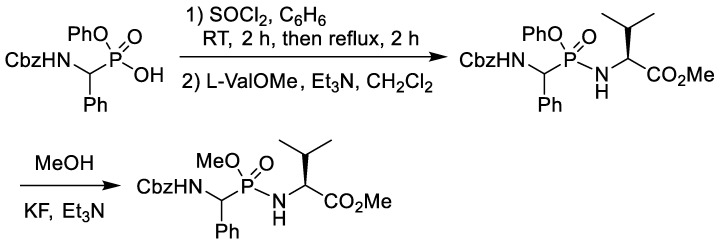
Synthesis of phosphonopeptide and its transesterification.

*O*-Phenyl phosphonamidates have been designed to bind covalently by nucleophilic substitution to the serine residue in the active site of serine proteases. Phosphonodipeptide with *O*-phenyl on the phosphorus atom was synthesized from phenyl *N*-Cbz 1-amino(phenyl)methylphosphonochloridate and methyl l-valinate. The stability of the phosphonamidates as a model of phosphonopeptides in aqueous solutions and their selectivity in the reaction against alcohols vs. thiols proved that they constituted a class of potential inhibitors of serine proteases and valuable tools to study the mechanism of inhibition. The transesterification of the phosphonopeptide phenyl ester with MeOH in the presence of Et_3_N/KF gave the corresponding phosphonopeptide methyl ester (Scheme 8) [34].

By using similar synthetic method, this research group also prepared phosphonodipeptides as new inhibitors of leucine aminopeptidase [35] and investigated the influence of the nature of the *N*-terminal functional group on their hydrolysis [36].

**Scheme 9:**
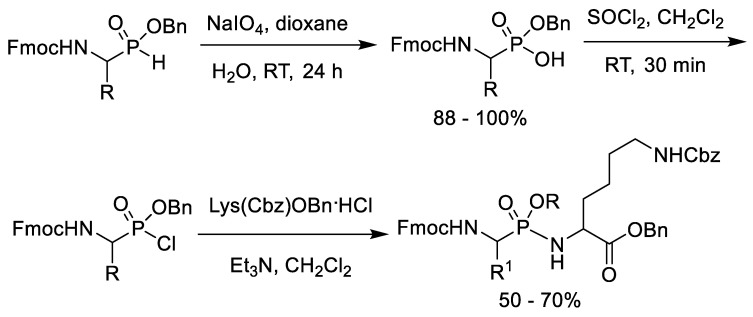
Synthesis of *N*-Fmoc protected phosphonopeptides.

Besides Cbz protecting group, Fmoc group was also used in the synthesis of phosphonopeptides. Benzyl hydrogen α-(9-fluorenylmethoxycarbonyl(Fmoc)amino)alkylphosphonates were obtained by the sodium metaperiodate oxidation of the corresponding phosphinates and converted to the phosphonochloridates, which were coupled with *N*^ε^-protected lysine benzyl ester to afford phosphonodipeptides containing a lysine residue (Scheme 9) [37].

**Scheme 10:**
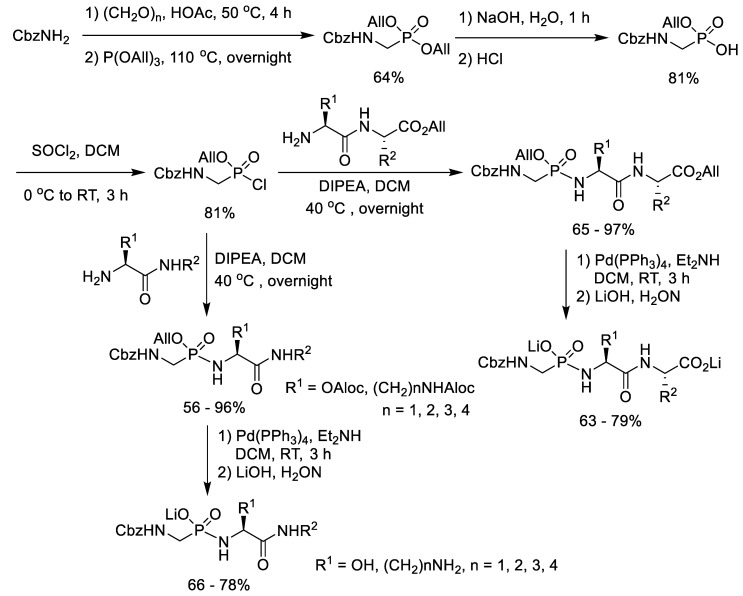
Synthesis of phosphonopeptides from diallyl *N*-Cbz aminomethylphosphonate.

Cramer and Klebe described a procedure for the synthesis and purification of functionalized phosphonopeptides that were able to generate inhibitors for the metalloprotease thermolysin for use in biophysiological experiments. The method utilized an allyl ester/allyloxycarbonyl(Aloc) protection strategy and showed advantage of a fast and effective solid-phase purification step. They first prepared diallyl *N*-Cbz aminomethylphosphonate and transformed it into allyl *N*-Cbz aminomethylphosphonochloridate, which was further reacted with dipeptide allyl esters or amino amide derivatives. After deprotection under the catalysis of Pd(PPh_3_)_4_ followed by treatment with LiOH, phosphonopeptide lithium salts were obtained (Scheme 10). By using the strategy, they synthesized a series of highly polar phosphonopeptide inhibitors with amino- and hydroxy-functionalized side chains in excellent purity [38].

**Scheme 11:**
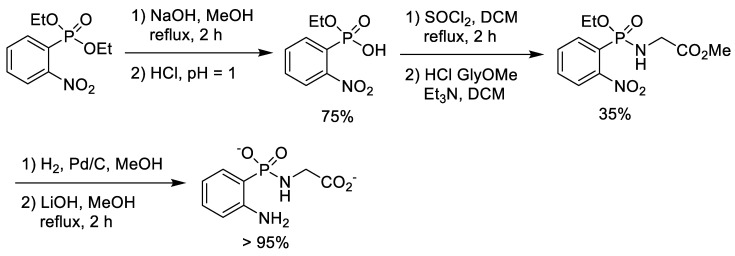
Synthesis of β-phosphonopeptide containing aromatic *N*-terminal amino group.

To search for effective and stable inhibitors of cytosolic leucine aminopeptidase, a β-phosphonopeptide containing aromatic *N*-terminal amino group was designed and synthesized. Diethyl 2-nitrophenylphosphonate was prepared and converted to the corresponding phosphonochloridate, which was coupled with methyl glycinate hydrochloride followed by reduction and basic hydrolysis, affording β-phosphonopeptide. The decrease in basicity of the terminal amino moiety of the β-phosphonopeptide resulted in satisfactory improvement of hydrolytic stability of the P−N bond. However, it did not exhibit inhibition activity up to millimolar concentration in enzymic assays towards leucine aminopeptidase possibly because diminishing the basic character of the terminal amino group resulted in a change of its affinity towards the zinc ions in the aminopeptidase. On the other hand, the decrease of the inhibition activity might also be ascribed to the steric constrain induced by the planar phenyl ring (Scheme 11) [39].

### 2.4. Chlorination of Alkyl Phosphonic Acid Monoesters with Oxalyl Chloride

**Scheme 12:**
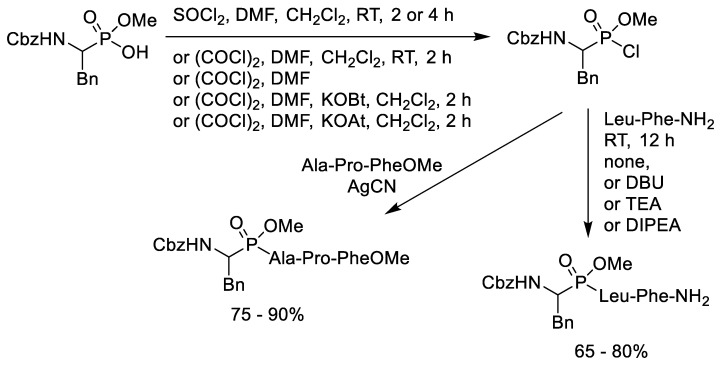
Synthesis of phosphonopeptides under mild conditions.

Moroder’s research group tested different chlorination conditions and found that oxalyl chloride-mediated preparation of phosphonochloridates allowed the improvement of the synthesis of phosphonopeptides, in comparison with thionyl chloride. A catalytic amount of *N*,*N*-dimethylformamide (DMF) promoted the chloridation. Particularly if AgCN was used as catalyst or upon conversion of the corresponding phosphonochloridates into their 7-aza-1-hydroxybenzotriazole (HOAt) ester, the yields of phosphonopeptides even reached 90% (Scheme 12) [24]. When peptide hydrochlorides were utilized as amino components triethylamine or diisoproylethylamine was added as base. However, triphosgene was an inefficient chloridating reagent. Furthermore, the Mukaiyama procedure failed completely in the present case. The mixed anhydrides of aminoalkylphosphonic acid monoesters with pivaloyl chloride generated, but affording exclusively to the *N*-pivaloylpeptide derivatives instead phosphonopeptides, despite the steric bulk of the *tert*-butyl group [24].

Various methods have been explored for the convergent synthesis of phosphonopeptides via phosphonochloridates. PCl_5_ is an effective reagent for the conversion of diethyl and diisopropyl phosphonates to the corresponding phosphonochloridates in the absence of complex functionality. Subsequent reaction with amino and peptide esters generated the phosphonopeptides in good yields. However, a milder method using oxalyl chloride to generate the phosphonochloridates from phosphonic monoesters was required in the presence of more complex functionality. Aminolysis of the dimethyl phosphonates or basic hydrolysis of diethyl phosphonates generated the requisite phosphonic monoesters for the preparation of phosphonochloridates [40].

**Scheme 13:**
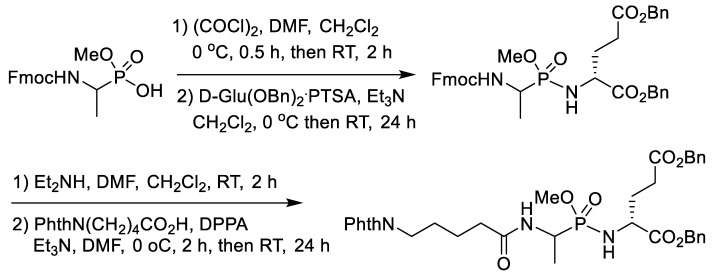
Synthesis of phosphonopeptides as potential immunomodulators.

New muramyl dipeptide (MDP) analogs related to LK 423 as potential immunomodulators was synthesized by coupling of methyl *N*-Fmoc 1-aminoethylphosphonochloridate and dibenzyl d-glutamate toluenesulfonate, followed by basic deprotection and coupling with 5-phthalimidopentanoic acid. The dipeptide part of the lead compound was modified by introducing a phosphonamidate bond instead of the amide bond between l-alanine and d-glutamic acid (Scheme 13) [25].

The synthesis of different muramyl dipeptide analogue LK 415 derivatives as potential immunomodulators was reported. The d-alanine and d-isoglutamine of LK 415 were replaced by their phosphonic analogues l,d-phosphonoalanine [H_2_NCHMeP(O)(OMe)_2_] and H_2_NCH[CH_2_CH_2_P(O)(OEt)_2_]COCH_2_Ph, yielding the LK 415 analogues *N*-[2-[2-[(1-adamantylcarbonyl)amino]ethoxy]acetyl]-NHCHMeP(O)(OMe)-d-Glu(OEt)_2_ and *N*-[2-[2-[(1-adamantylcarbonyl)amino]ethoxy]acetyl]-l-Ala-NHCH[CH_2_CH_2_P(O)(OEt)_2_]COCH_2_Ph, respectively [41].

**Scheme 14:**
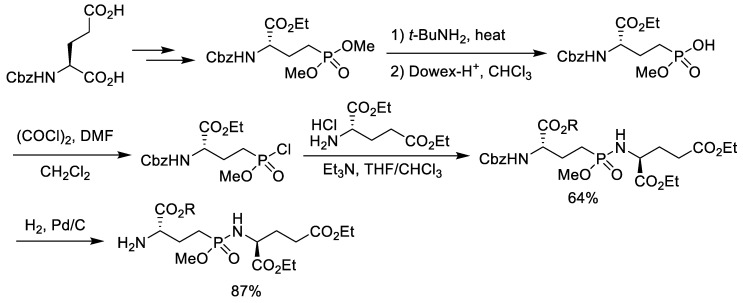
Synthesis of γ-phosphonopeptides.

To prepare phosphorus analogues of γ-glutamyl peptide, *N*-Cbz l-glutamic acid was first transformed to the corresponding dimethyl phosphonate. After aminolysis and chlorination it was transformed to the corresponding phosphonochloridate, which was reacted with diethyl glutamate, affording γ-phosphonodipeptide in 64% yield. After hydrogenolysis, *N*-terminal free γ-phosphonopeptide was obtained in 87% yield. However, the coupling of the phosphonochloridate with diethyl 2-hydroxyglutarate generated γ-phosphonodepsipeptide in only 6.7% yield, indicating the strategy was not suitable for the synthesis of γ-phosphonodepsipeptide (Scheme 14) [42].

### 2.5. Chlorination of Alkyl Trimethylsilyl Phosphonites or Alkyl Phosphinates with Carbon Tetrachloride

**Scheme 15:**
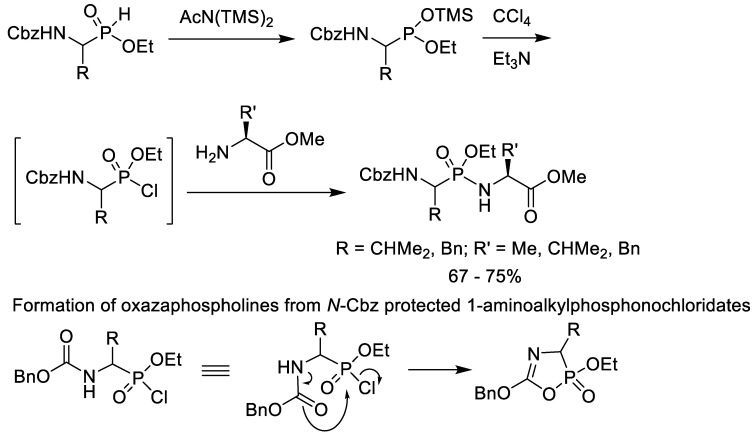
Synthesis of phosphonopeptides via chlorination of aminoalkylphosphonites.

To minimize the side reactions (formation of oxazaphospholines) of *N*-Cbz protected 1-aminoalkylphosphonochloridates, a convenient method for the synthesis of phosphonopeptides was described. *N*-Cbz protected 2-aminoalkylphosphinates were converted to their trimethylsilyl phosphonite tautomers with the treatment with bis(trimethylsilyl)acetamide. They were oxidized with CCl_4_ to generate the corresponding phosphonochloridates as intermediates, which reacted with amino acid esters to give rise to the desired phosphonopeptides. The oxidative activation was carried out in the presence of the amine nucleophiles so that stoichiometric formation of the phosphonochloridates were avoided and side reactions were minimized (Scheme 15) [26].

**Scheme 16:**
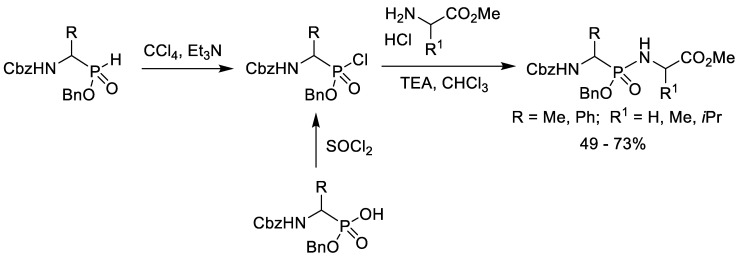
Synthesis of phosphonopeptides from aminoalkylphosphinates.

Phosphonylation of amino acid esters with *N*-Cbz 1-aminoalkylphosphonochloridates was a general method for the preparation of phosphonopeptides containing a phosphonamidate bond. *N*-Cbz 1-aminoalkylphosphonochloridates were prepared from both *N*-Cbz 1-aminoalkylphosphinates with carbon tetrachloride in the presence of trimethylamine through the Atherton–Todd reaction and the corresponding *N*-Cbz 1-aminoalkylphosphonic acid monoesters with thionyl chloride (Scheme 16) [27]. 

### 2.6. Bromination of Alkyl Phosphinates with Bromine

**Scheme 17:**
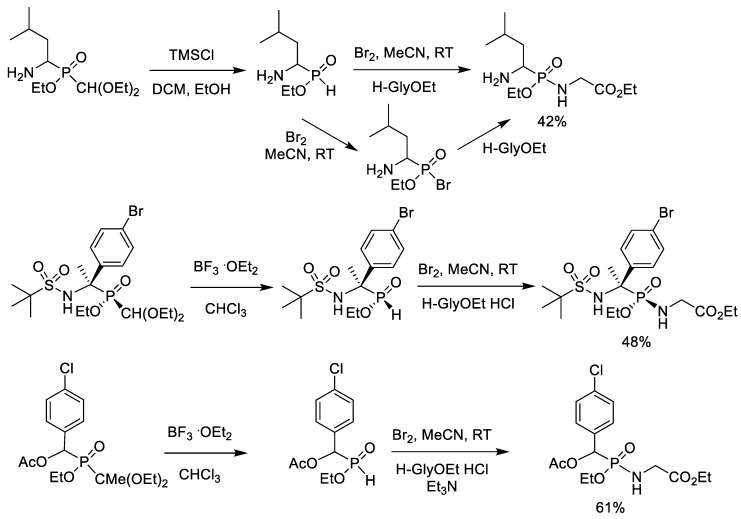
Synthesis of phosphonopeptides via phosphonobromidates.

Yao and Yuan developed a general and efficient one-pot procedure for converting 1,1-diethoxyalkylphosphinates into phosphonates or phosphonamides by the use of bromine. For α-aminoalkylphosphinates, the transformation could be realized without the protection of the amino group. Enantiopure phosphinate reacted stereospecifically with bromine and subsequently couples with nucleophile to generate the corresponding optically active derivatives with retention of configuration at the phosphorus center (Scheme 17) [28]. The developed method was applied in the synthesis of phosphonopeptides.

**Scheme 18:**
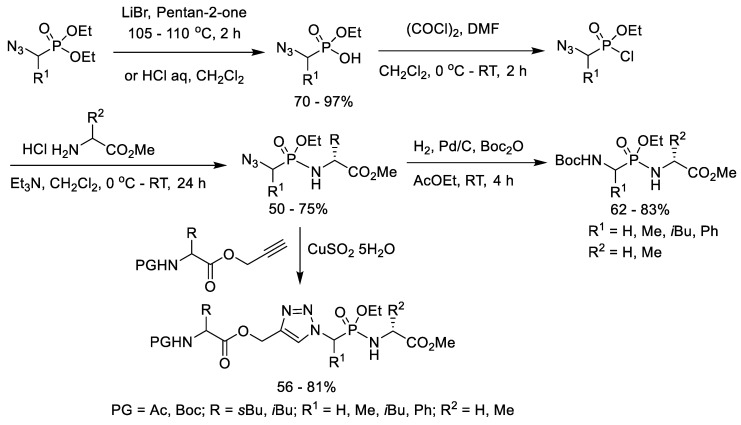
Synthesis of phosphonopeptides and triazole-containing phosphonopeptides via 1-azidoalkylphosphonochloridates.

A new method for the synthesis of *tert*-butoxycarbonyl(Boc)-protected phosphonopeptides was developed with diethyl 1-azidoalkylphosphonates as starting materials. After partial hydrolysis and treatment with oxalyl chloride under the catalysis of DMF, diethyl 1-azidoalkylphosphonates were transformed to ethyl 1-azidoalkylphosphonochloridates, which were coupled with amino ester hydrochlorides to afford azidophosphonodipeptides. They were further converted *N*-Boc protected phosphonodipeptides under hydrogenolysis in the presence of Boc_2_O in ethyl acetate (Scheme 18) [43].

The click reaction of azidophosphonodipeptides and *N*-protected leucine/isoleucine propargyl esters with different protecting group (PG) under the catalysis of copper sulfate pentahydrate, affording triazole-containing phosphonopeptides (Scheme 18) [44].

## 3. Synthesis of Phosphonopeptides with Coupling Reagents

Condensation of *N*-protected amino acids or peptides with amino/peptide esters is a general method for peptide synthesis. The method has also been applied in the synthesis of phosphonopeptides with carbodiimides, *N,N*′-dicyclohexylcarbodiimide (DCC) and *N,N*′-diisopropylcarbodiimide (DIC), diphenylphosphoryl azide (DPPA), and benzotriazole-1-yl-oxytripyrrolidinophosphonium hexafluorophosphate (PyBop) as coupling reagents, respectively.

**Scheme 19:**
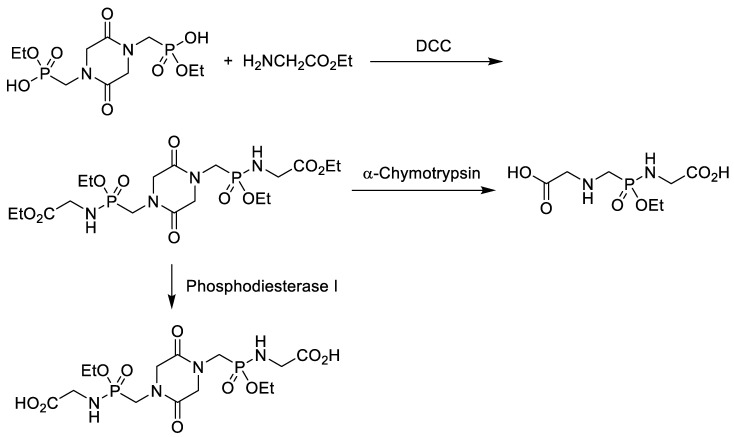
Synthesis of phosphonopeptides with *N,N*’-dicyclohexylcarbodiimide (DCC) as a coupling reagent.

The diketopiperazine-derived diphosphonic monoethyl ester was condensed with ethyl glycinate to afford diketopiperazine-derived phosphonodipeptide. Strict selectivity was observed upon enzyme-catalyzed hydrolysis. Enzyme α-chymotrypsin catalyzed the hydrolysis of the diketopiperazine ring and the ethyl ester group to give the free acids HO_2_CCH_2_NHCH_2_P(O)R^1^NHCH_2_CO_2_H. While phosphodiesterase I catalyzed the hydrolysis of only the ethyl ester group (Scheme 19) [45].

**Scheme 20:**
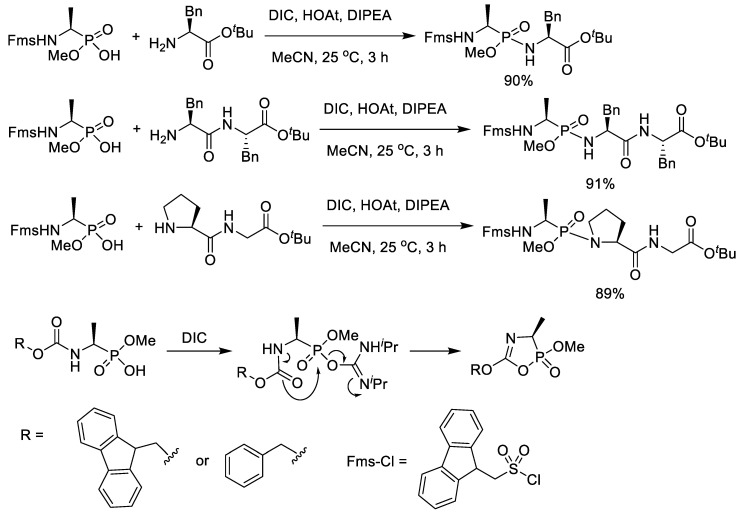
Synthesis of phosphonopeptides with *N,N*’-diisopropylcarbodiimide (DIC) as a coupling reagent.

Kitamura’s group developed a sulfonamide-based protecting group, (9*H*-fluoren-9-yl)methanesulfonyl (Fms). It was used in a similar way to the well-established Fmoc protecting group. It was demonstrated in the successful formation of a phosphonamidate between an *N*-Fms-protected α-aminoethylphosphonic monoester with amino or dipeptide esters, including (*S*)-phenylalanine *tert*-butyl ester (H-Phe-O*t*Bu), H-Pro-Gly-O*t*Bu, and H-Phe-Phe-O*t*Bu, without formation of oxazaphospholine byproduct, which was a serious problem associated with the Fmoc and Cbz protecting groups during the formation of the phosphonamidate bond in both coupling reagent method [46] and phosphonochloridate method [24] (Scheme 20).

**Scheme 21:**
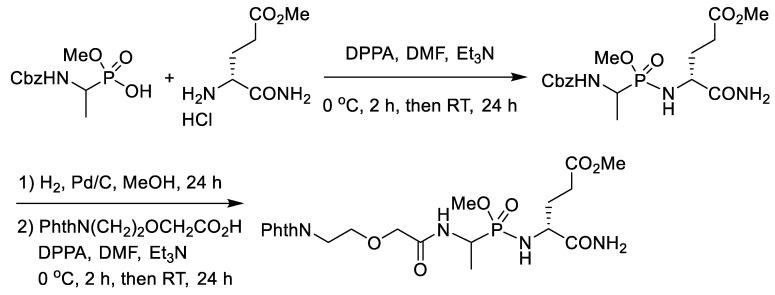
Synthesis of phosphonopeptides with diphenylphosphoryl azide (DPPA) as coupling reagent.

MDP phosphorus analogs related to LK 423 as potential immunomodulators were synthesized by coupling of methyl 1-(*N*-benzyloxycarbonyl)aminoethylphosphonate and methyl d-isoglutamate hydrochloride with DPPA as coupling reagent, followed by hydrogenolysis and coupling with 2-(2-phthalimidoethoxy)acetic acid. The dipeptide part of the lead compound was modified by introducing a phosphonamidate isostere instead of the amide bond between l-alanine and d-isoglutamine (Scheme 21) [25].

**Scheme 22:**
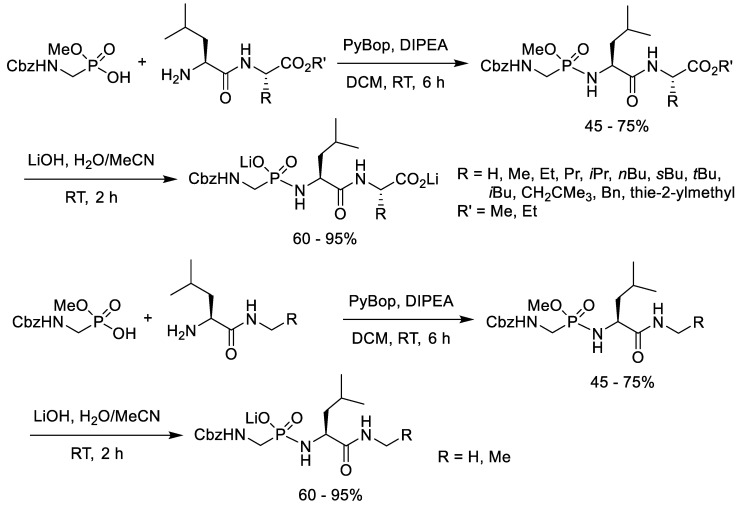
Synthesis of phosphonopeptides with benzotriazole-1-yl-oxytripyrrolidinophosphonium hexafluorophosphate (PyBop) as a coupling reagent.

To investigate the ligand–protein binding thermodynamics of the hydration waters of ligand-thermolysin complexes and hydrophobic binding, various thermolysin phosphonopeptide inhibitors were synthesized via the PyBop coupling of *N*-Cbz-protected aminomethylphosphonic methyl monoester with dipeptide esters or amino amides. The thermodynamic study provided important understanding on the water role in ligand–protein binding (Scheme 22) [47,48,49].

## 4. Synthesis of Phosphonopeptides Catalyzed by Enzyme

**Scheme 23:**
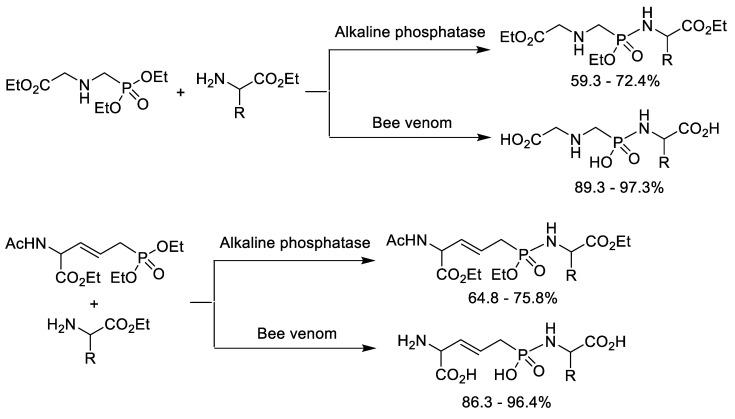
Synthesis of α- and δ-phosphonopeptides.

Natchev investigated the enzyme-catalyzed synthesis of phosphonopeptides. The alkaline phosphatase-catalyzed reaction of 1-aminomethylphosphonate and amino acid esters gave rise to the corresponding phosphonodipeptide esters, while the bee venom-catalyzed reaction of 1-aminomethylphosphonate and amino acid esters generated free phosphonodipeptides (Scheme 23) [50].

**Scheme 24:**
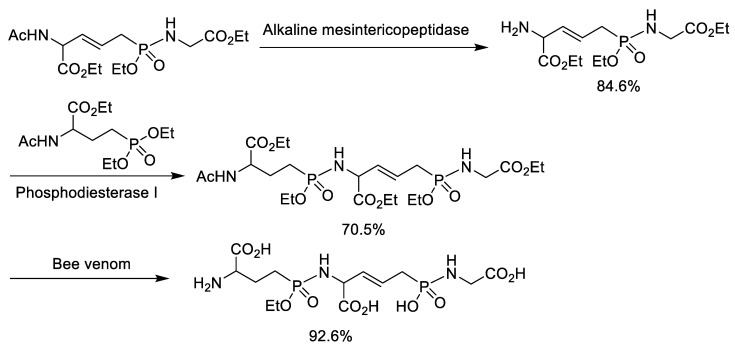
Synthesis of phosphonopeptides composing of γ- and δ-aminoalkylphosphonic acid residues.

Alkaline mesintericopeptidase selectively hydrolyzed *N*-acetyl protection group to afford *N*-terminal free phosphonodipeptide, which was further reacted with phosphonate under the catalysis of phosphodiesterase I to give protected phosphonotripeptide. After deprotection under the catalysis of bee venom, free phosphonotripeptide was obtained (Scheme 24) [50].

**Scheme 25:**
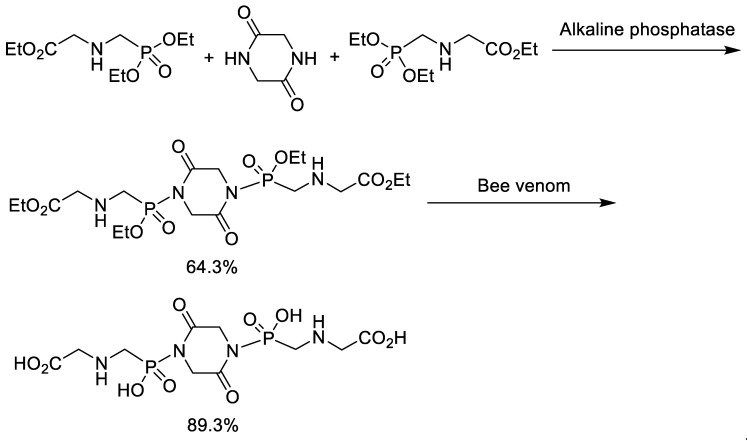
Synthesis of phosphonopeptides containing a cyclodipeptide core.

The alkaline phosphatase-catalyzed reaction of cyclic dipeptide with two molecules of 1-aminomethylphosphonate generated the corresponding phosphonotetrapeptide ester with a cyclic dipeptide moiety in the center. The bee venom-catalyzed hydrolysis of the protected phosphonotetrapeptide realized synthesis of free phosphonopeptide (Scheme 25) [50].

## 5. Synthesis of Phosphonopeptides via Phosphonochloridite Followed by Oxidation

**Scheme 26:**
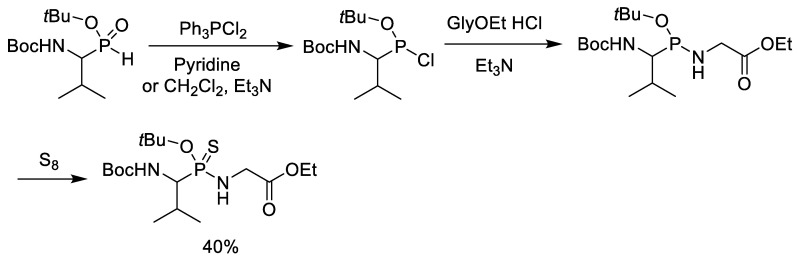
Synthesis of thiophosphonopeptides.

Hammer’s group developed a new strategy to synthesize phosphonopeptides via coupling of phosphonochloridites and amino esters and subsequent sulfur oxidation. They used *N*-Boc protected 1-aminoalkylphosphinate as starting material and converted it into the corresponding phosphonochloridite with dichlorotriphenylphosphorane. The phosphonochloridite was further coupled with ethyl glycinate hydrochloride followed by sulfurization with sulfur, affording phosphonothiopeptide in one-pot activation-coupling-oxidation procedure. They mentioned that the phosphonochloridites were more active species than the corresponding phosphonochloridates (Scheme 26) [51].

**Scheme 27:**
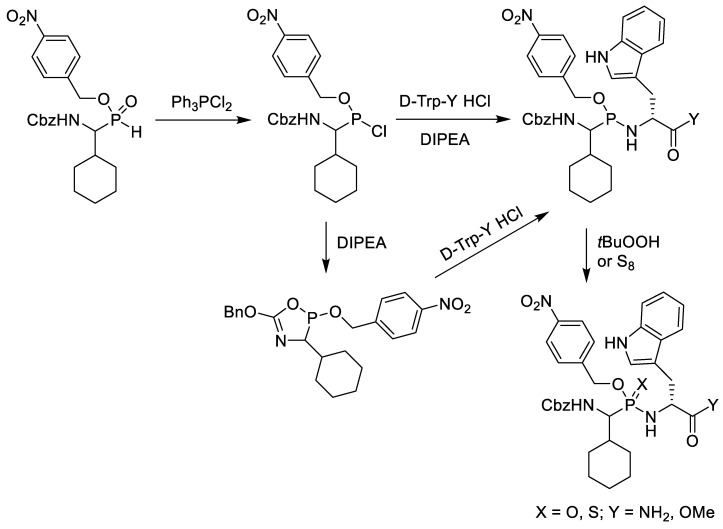
Synthesis of phosphonopeptides via phosphonochliridites.

Rushing and Hammer further applied the new strategy in the synthesis of *N*-Cbz protected phosphonodipeptides and phosphonothiopeptides using their P(III) one-pot activation-coupling-oxidation procedure. After treatment with dichlorophosphorane, *N*-Cbz protected 1-amino(cyclohexyl)methylphosphinate was transformed to the P(III) intermediate, *N*-Cbz protected 1-amino(cyclohexyl)methylphosphonochloridite, which was reacted with d-tryptophan derivatives in the presence of diisopropylethylamine (DIPEA) followed by oxidation with tertial butyl peroxide and sulfur, affording phosphonopeptides and phosphonothiopeptides, respectively. It was found that base DIPEA could cyclize the *N*-Cbz 1-aminomethylphosphonochloridite into 5-(benzyloxy)-2,3-dihydro-1,4,2-oxazaphosphole. However, it was further reacted with d-tryptophan derivatives to generate the same intermediate phosphinamides in the reaction system (Scheme 27) [52].

## 6. Synthesis of Phosphonopeptides via Pseudo Four-Component Condensation Reaction

**Scheme 28:**
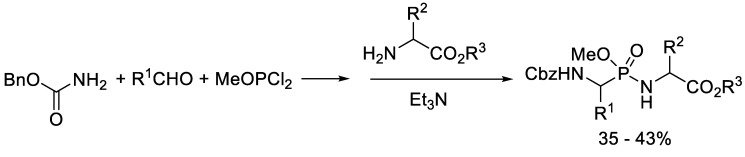
Synthesis of phosphonopeptides via pseudo four-component condensation.

Previously the Mannich-type reaction of benzyl carbamate, aldehydes, and trialkyl phosphites in acetyl chloride gave rise to *N*-Cbz-1-aminoalkylphosphonates [31]. We further developed the method as a pseudo-four-component condensation reactions for synthesis of *N*-Cbz 1-aminoalkylphosphonic acid derivatives, including phosphonamidates [53], mixed esters [54,55], and phosphonodepsipeptides [56,57,58,59]. The four-component condensation was applied as a direct method for the preparation of phosphonopeptides in construction of 1-aminoalkylphosphonic acids simultaneously formed the phosphonamidate bond. Using this method, phosphonopeptides were prepared in acceptable yields directly from simple and commercially available chemicals in one-pot reactions of benzyl carbamate, aldehydes, and methyl dichlorophosphite, followed by aminolysis with amino acid esters (Scheme 28) [60].

**Scheme 29:**
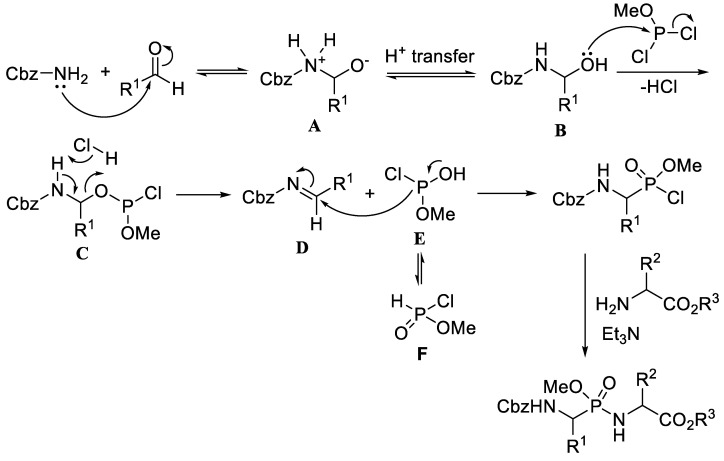
Mechanism for the synthesis of phosphonopeptides via pseudo four-component condensation.

The pseudo four-component condensation reaction mechanism is proposed as following. Benzyl carbamate first nucleophilically attack aldehydes followed by proton transfer to generate α-amino alcohols **B**, which undergo a nucleophilic substitution with methyl dichlorophosphite on the phosphorus atom, yielding chlorophosphites **C**. Chlorophosphites **C** undergo an elimination to give rise imines **D** and chlorophosphonous acid **E** in the presence of HCl. Chlorophosphonous acid **E** can exist as **E** and H-phosphonic chloride **F**. However, chlorophosphonous acid **E** is efficient species to nucleophilically add to the C=N bond or protonated C=N bond of imines **D** to produce the phophonochloridates as intermediates. After aminolysis with amino esters, the desired phosphonopeptides were obtained (Scheme 29) [54,57].

The pseudo four-component condensation method is an efficient and convergent strategy for the synthesis of phosphonopeptides from simple and commercially available chemicals, such as, benzyl carbamate, aldehydes, methyl dichlorophosphite, and amino acid esters. Compared with other strategies, the strategy combines the construction of α-aminoalkylphosphonic acids and the formation of the phosphonamidate bond in a one-pot mode. However, other reported methods construct α-aminoalkylphosphonic acid derivatives first and then form the phosphonamidate bond.

## 7. Synthesis by Nucleophilic Addition

**Scheme 30:**
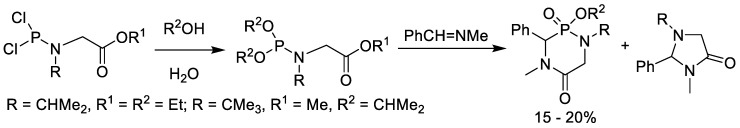
Synthesis of cyclophosphonopeptides via nucleophilic addition.

Gololobov and Nesterova synthesized cyclic phosphonodipeptides via the annulation of *N*-benzylidenemethylamine and alkyl *N*-(dialkoxyphosphanyl)-*N*-alkylglycinates, which were prepared from alkyl *N*-(dichlorophosphanyl)-*N*-alkylglycinates with alcohols R^2^OH. The annulation was a sequence of P-nucleophilic addition and intramolecular aminolysis accompanying an N-nucleophilic addition followed by intramolecular aminolysis to yield imidazolidin-4-ones as byproducts (Scheme 30) [61].

## 8. Conclusions

Phosphonopeptides are a class of phosphorus analogues of peptides containing a tetrahedral phosphonamidate bond, which can mimic the transition state of amide hydrolysis. Thus, phosphonopeptides have been widely applied as enzyme inhibitors and antigens for catalytic antibodies. They have been utilized as antibacterial agents as well. Various synthetic methods of phosphonopeptides have been developed, mainly including phosphonylation of amino/peptide esters with *N*-protected aminoalkylphosphonochloridates, condensation of *N*-protected phosphonic monoesters and amino/peptide ester with coupling reagents and enzyme, phosphinylation of amino/peptide esters with *N*-protected aminoalkylphosphonochloridites followed by oxidation. Pseudo four-component condensation is a convergent and efficient strategy for synthesis of phosphonopeptides from simple starting materials. The synthetic methods of phosphonopeptides will show wide application in understanding biological function of biomacromolecules and development of medicines in the future.
